# Analysis and Modeling of Aged SAC-Bi Solder Joints Subjected to Varying Stress Cycling Conditions

**DOI:** 10.3390/ma16020750

**Published:** 2023-01-12

**Authors:** Minghong Jian, Sa’d Hamasha, Ali Alahmer, Mohammad Hamasha, Xin Wei, Mohamed El Amine Belhadi, Khozima Hamasha

**Affiliations:** 1 Department of Industrial and Systems Engineering, Auburn University, Auburn, AL 36849, USA; 2 Department of Mechanical Engineering, Faculty of Engineering, Tafila Technical University, Tafila 66110, Jordan; 3 Department of Industrial Engineering, Faculty of Engineering, The Hashemite University, Zarqa 13133, Jordan; 4 Department of Basic Scientific Sciences, Al-Huson University College, Al-Balqa Applied University, Al-Salt 19117, Jordan

**Keywords:** fatigue, reliability, SAC solder alloys, varying stresses, Bismuth, modeling

## Abstract

Solder joints are subjected to varied stress cycle circumstances in the electronic packaging service life but are also influenced by aging. There has been limited investigation into the influence of aging and varying cycles on SnAgCu-Bi (SAC-Bi) solder joint fatigue. Cyclic fatigue tests were performed on solder joints of several alloys, including SnAgCu (SAC305), SnAgCu-Bi (SAC-Q), and SnCu-Bi (SAC-R). Individual solder joints were cycled under varying stress levels, alternating between mild and harsh stress levels. At least seven samples were prepared for each alloy by alternating between 25 mild stress (MS) cycles and three harsh stress (HS) cycles until the solder joint broke off. The impact of aging on Bi-doped solder joints fatigue under varied amplitude stress was examined and predicted for 10 and 1000 h under 125 °C. Because of the “Step-up” phenomenon of inelastic work, a new fatigue model was developed based on the common damage accumulation (CDA) model. The experimental results revealed that aging reduced the fatigue life of the tested solder alloys, particularly that of SAC305. According to the CDA model, all solder alloys failed earlier than expected after aging. The proposed model uses the amplification factor to assess inelastic work amplification after switching between the MS and HS cycles under varying stress amplitude conditions. The amplification factor for the SAC-Bi solder alloys increased linearly with fracture initiation and substantially followed crack propagation until the final failure. Compared with existing damage accumulation models, the proposed fatigue model provides a more accurate estimation of damage accumulation. For each case, the cut-off positions were examined. The SAC-Q amplification factor increased linearly to 83% of its overall life, which was much higher than that of SAC305 and SAC-R. This study identified three distinct failure modes: ductile, brittle, and near intermetallic compound (IMC) failure. It was also observed that SAC-Q with an organic solderability preservatives (OSP) surface finish was more susceptible to brittle failure owing to the excessive brittleness of the alloy material.

## 1. Introduction

Eutectic tin-lead (Sn-Pb) solder alloys are frequently employed in the electronics packaging industry. Because of the environmental and health risks of lead, it is being phased out and replaced by a new semi-eutectic solder alloy. Microalloying with nickel (Ni), bismuth (Bi), antimony (Sb), indium (In), and other elements could enhance the performance of solder alloys [1]. Although extensive studies have been performed on the mechanical and thermal characteristics, including fatigue of various solder alloys using accelerated test conditions, the reliability of solder joints in real service requires further investigation to be better understood. Because the applied mechanical stresses on solder joints frequently change under real conditions, it is necessary to test solder alloys under accelerated testing conditions. 

Kanchanomai et al. [2] observed that microalloying with Bi increased fatigue life in a strain-controlled test. Al Athamneh et al. [3] examined the fatigue performance of SAC305 and SAC-Bi solder joints by considering the aging effects. Regardless of stress or aging conditions, SAC-Bi was shown to have a longer fatigue life than SAC305. Other analyses have scrutinized the shear fatigue characteristics of SAC-based micro-alloys with Bi, Ni, and Sb under various organic solderability preservatives (OSP) and immersion silver (ImAg) surface finishes [1]. It was observed that the greater the Ag content, the better the fatigue and shear strengths achieved [4]. Xu et al. [5,6] studied the fatigue characteristics of SAC and SnPb-based alloys under isothermal fatigue conditions. Shear fatigue tests were conducted using plastic ball grid array (PBGAs) to evaluate their reliability over a wide strain range. The fatigue constants derived from the Coffin-Manson model provide information regarding the mechanical properties. SAC305 outperformed SAC105 and SnPb in specific strain ranges. Hamasha et al. [7] evaluated the reliability of SAC-based alloys under varying stress situations. The findings indicated that the fatigue resistance of solder joints changes with stress amplitude by a factor of −6.5 as the ductility exponent. Aging causes considerable deterioration in mechanical parameters such as stress–strain, working per cycle, and fatigue life [8]. The change in the mechanical properties of SAC-based materials is due to the formation of Cu_6_Sn_5_ on the copper pad [9]. The influence of aging on the cyclic stress–strain diagram of Bi-SAC alloys was investigated by Chowdhury et al. [10]. The authors found that the Bi-based alloys exhibited smaller plastic strain ranges and much higher peak stresses than the SAC-based alloys. Consequently, the function of Bi in inhibiting dislocation movement within the Sn matrix and the distribution of dominating intermetallic compound (IMC) particles have been attributed to the improvement in cyclic stress–strain characteristics [11]. SAC alloys with Ni or Bi exhibit improved thermal fatigue life, according to Lee et al. [12]. Cai et al. found that adding elements such as Bi, Ni, In, Mn, Ce, and Mg to typical SAC alloys resulted in a more stable behavior with less stress–strain and creep mechanical property loss during aging [13]. Dalton et al. [14] examined the impact of induced strain on the reliability of the third-generation alloys SAC387, Innolot, and Castin (Sn-Ag2.5-Cu0.8-Sb0.5). A high amount of strain was detected at the high-temperature range.

Several fatigue models have been developed to forecast the fatigue life of solder joints. Damage accumulation, creep damage, plastic strain, and energy-based models are some of the models employed. Miner’s damage accumulation model uses a linear relationship to estimate fatigue life. The accumulated damage in each cycle is independent of the load [15]. Corten and Dolan [16] presented a nonlinear accumulation damage model in which cracks propagated at both high and low-stress amplitudes. Further studies have examined the impact of a low-stress amplitude below the fatigue limit while considering the initial damage. A low-stress amplitude was observed to contribute to damage accumulation depending on the preceding damage history [17]. Zhu et al. [18] suggested a dynamic Corten- Dolan equation. Kariya et al. [19] proposed a partitioning equation based on the Manson method. This approach divides the fully reversed inelastic strain range into four general components: creep, plasticity, and reversal of the tensile strain components. Shi et al. [20] proposed a modified Morrow energy model. Unexpectedly, the material parameter should decrease as the temperature increases in order to preserve the consistency of ductility coefficient. In addition, as the temperature increases, the flow stress of the material decreases.

Based on a review of the relevant literature, two limitations emerge:

1. Limited studies have investigated how aging affects the stress of individual SAC-Bi solder joints when exposed to varying stress conditions. In general, aging cannot be completely prevented throughout the service life of a solder joint. Few investigations have been conducted on the effect of Bi doping on the fatigue life of SAC solder joints under varying stress amplitude conditions after aging.

2. A typical damage accumulation model cannot estimate the fatigue life of a solder joint under cycling stress. Consequently, an improved damage accumulation model is required to assess the reliability of solder joints under varied stress amplitudes.

In order to overcome the prior limitations, this article: (i) The effect of aging time and varying stress amplitude conditions on the fatigue life of SAC-Bi weld joints was investigated. Single SAC-Bi solder joints were aged for 10 h and 1000 h at 125 °C and cycled to complete failure under varying stress amplitudes. (ii) A reliability assessment was conducted on aging individual SAC-Bi solder joints at various stress amplitudes. (iii) The fatigue life of aged SAC-Bi solder alloys was compared under varying stress amplitude conditions. (iv) Precipitates and IMC layer analyses were performed on aged individual SAC-Bi solder joints to identify failure mechanisms under varying stress amplitude settings. (v) Comparison of the aging effect on failure modes by scanning electron microscope (SEM) microstructure analysis. Finally, (v) a novel damage accumulation model is proposed to assess the fatigue life of SAC-Bi under various stress amplitude situations.

The results indicated that SAC305 and SAC-R had extremely similar cut-off points, indicating that SAC-R has competitive fatigue resistance by replacing 3% Ag with 2.46% Bi. The combination of Ag and Bi in SAC-Q solder alloy exhibited the highest cut-off point, demonstrating the superior fatigue resistance under varying stress cycling conditions.

## 2. Individual Solder Materials, Sample Preparation, and Methods

Individual solder joints are typically implemented on customized printed circuit boards (PCBs) with OSP surface treatment. Therefore, testing with individual solder joints should provide a more accurate representation of the solder joint performance. The IMC layer is a critical component in determining the fatigue life of the solder joints.

### 2.1. Experimental Design

**Figure 1:**
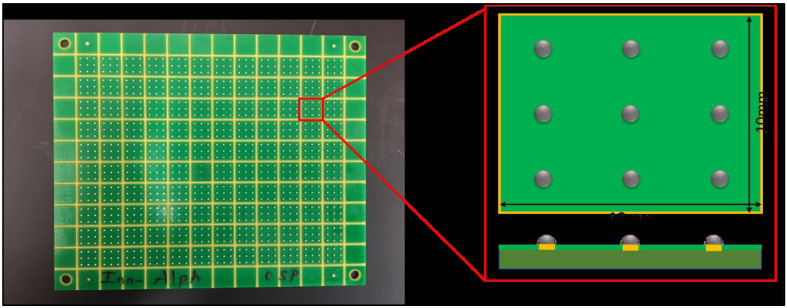
Test vehicle and individual solder joint.

In this study, individual SAC-Bi solder joints, SAC305, SAC-Q, and SAC-R, were subjected to cycling with fluctuating stress amplitude. Table 1 lists the chemical composition of each alloy. In all the tests, SAC305 was used as a baseline to examine the performance of various solder alloy systems. SAC-R has been developed as an economical substitute for SAC305 in the market since it contains low-priced Bi (~$10/lb) rather than costly Ag (~$270/lb). The high-priced SAC-Q solder with high Ag and Bi content is expected to provide more reliability. Figure 1 demonstrates the test vehicle. It consists of nine solder joints (30 mil diameters) installed on FR-4 PCBs (10 mm × 10 mm, nine inputs/outputs (I/Os), and a 3 mm pitch) with an OSP surface finish.

After designing the PCBs and materials for the doped solder joints, a stencil printing machine DEK Galaxy (ASM Assembly Systems, USA Inc., Rolling Meadows, IL, USA) was used. The tacky flux was first printed on the copper pads through the apertures of a stencil made of stainless steel. S Subsequently, diameter solder spheres of 0.762 mm were brushed on the tacky flux through another stencil with wider apertures. A quality check was performed to ensure that the amount, area, and placement of solder spheres were optimal. The entire assembly was reflowed in a nitrogen-gas atmosphere with less than 50 ppm O_2_ using an eight-zone Pyramax 100 N reflow oven. The reflow profile includes a 60-s preheat zone, a 120-s soak zone, and a 60-s reflow zone with a peak temperature of 245 °C and a cooling rate of 3.5 °C per second.

**Figure 2:**
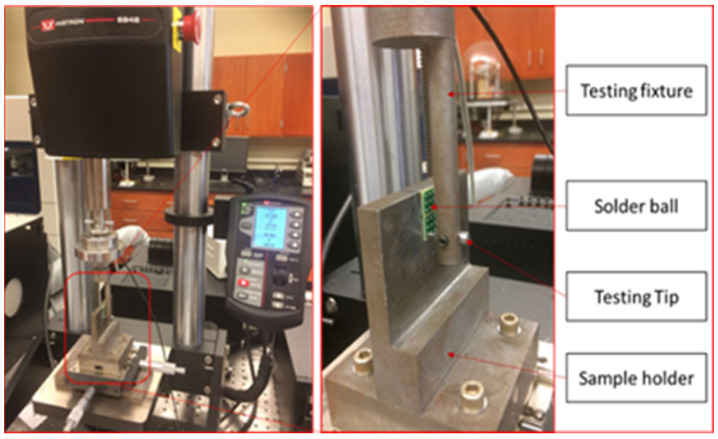
Instron 5948 MicroTester.

**Figure 3:**
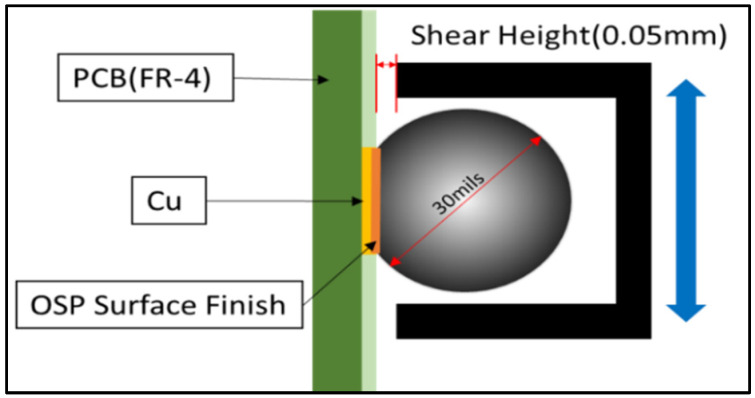
Cycling of solder joint.

The cyclic fatigue test was performed using an Instron 5948 (Instron-Materials Testing Equipment, Norwood, MA, USA)micromechanical tester, as displayed in Figure 2. It features a force range of 2 mN to 2 kN and extension resolution of 20 nm. The number of cycles, stress–strain data, stiffness, and other characteristics were recorded using an Instron micromechanical test. A fixture was developed to hold the testing tip, and nine separate solder joints on the PCB test coupons were bonded to a strong sample holder. In Figure 3, the side view of the fixture shows the interaction of individual joints with the two sides (tip). The internal diameter of the tip was 40 mils, and it was positioned 0.05 mm away from the PCP surface. Individual solder joints are usually fastened to the fixture, and the testing fixture is cyclically moved to reach the set-up stresses. When the testing tip moved more than 0.6 mm during a stress-controlled cyclic fatigue test, it was considered a failure. The PCB was sheared away from solder joints.

### 2.2. Test Matrix

Before the cyclic fatigue test, the three solder alloys were evaluated, and each solder joint was aged at 125 °C for 10 and 1000 h. The applied stress on the solder joints was alternated between MS and HS, predefined based on a “single stress amplitude test”, as will be explained later. According to the fitted power equation, the predicted MS and HS amplitudes matched with 2500 and 300 typical life (cycles). Table 2 summarizes the MS and HS data. As shown in Table 3, at least seven samples of each solder alloy were evaluated by alternating 25 MS cycles with three HS cycles until the ultimate failure. Experiments were designed based on the concept of damage accumulation. In general, a switch from 25 MS amplitude cycles to three HS amplitude cycles is projected to result in 2% damage (25/2500 + 3/300 = 2%). Therefore, fifty alternations were expected during the test. However, all solder joints failed far sooner than expected, as will be discussed later.

## 3. Miner’s Model of Doped Solder Joints

**Figure 4:**
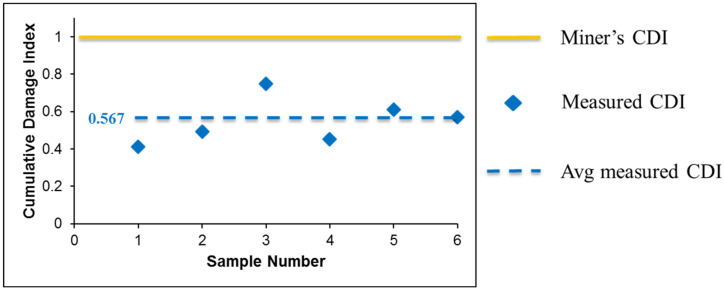
Breakdown of Miner’s rule.

Miner’s rule is a well-known theory for forecasting the fatigue life due to linear damage accumulation, and it provides the following index [21]:

(1) $$\mathrm{CDI}=\sum^{\;}\frac{n_{i}}{N_{i}}$$

where $n_{i}$ is the number of cycles, and $N_{i}$ is the amplitude *i.* According to Miner’s rule, the alloy fails at an index of 1. However, Miner’s rule collapses when the stress amplitude fluctuates. In the variable stress amplitude test, the solder joints of SAC305 were cycled 25 times at 18 MPa, and then three times at 27.6 MPa. This alternating continued until final failure, as shown in Figure 4. According to Miner’s rule, all samples were damaged in varying ranges from 40% to 80%, with an average of approximately 57%. However, all samples failed the test, indicating that CDI should be 1. As a result, Miner’s rule overrates the fatigue life of SAC 305 in varying amplitude tests. Additionally, some initial studies have reported that Miner’s rule does not work well under variable amplitudes [22,23,24,25,26,27], and a modified Miner’s model is obtained as follows, which defines the amplification factor f(i) [20,27]:

(2) $$1=\overset{s}{\underset{i}{\sum }}f\left(i\right)\frac{n_{mi}}{N_{m}}+\frac{n_{hi}}{N_{h}}$$

where $n_{mi}$ is the number of cycles at low amplitude, $N_{m}$ is the life at low amplitude, $n_{hi}$ is the number of cycles at high amplitude, $N_{h}$ denotes the life at high amplitude. The amplification factor is defined as the impact of high-stress cycles on the work per cycle in the subsequent low-stress cycles [22].

## 4. Results and Discussion

**Figure 5:**
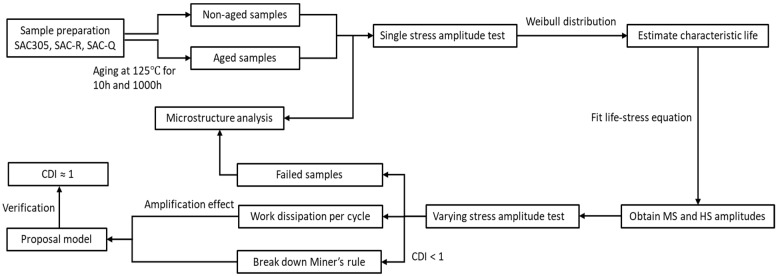
Block diagram of the of the experimental tests.

The obtained results are discussed in five distinct parts: an examination of the fatigue life, inelastic work, amplification of inelastic work and power fit equation, the proposed fatigue model in terms of a modified accumulative damage model, and microstructural analysis. Figure 5 shows the block diagram of the experimental tests. Part of the samples was aged to investigate the aging effect. Single stress amplitude tests were conducted to estimate the characteristic fatigue life using the Weibull distribution. These data were fitted in a power equation for further determining the MS and HS amplitudes applied in varying stress amplitude tests. A modified accumulative damage model was proposed based on the results of the work dissipation analysis. The microstructure of different samples was identified.

### 4.1. Characteristic Fatigue Life

The fatigue performance of solder alloys containing Bi was investigated by adopting the following strategy: first, under single-stress amplitude conditions without aging, then under single-stress amplitude conditions with aging, and finally, under varying stress with aging. One of the most critical aspects of a single stress amplitude is determining the MS and HS amplitudes matched with a typical life of 2500 and 300 cycles. Therefore, a single stress amplitude was applied for SAC305, SAC-Q, and SAC-R, and their ideal life in terms of cycles under each stress amplitude are presented in Table 4. At least seven individual solder joints were cycled to final failure under each single stress amplitude. After fitting the fatigue life of the seven individual solder joints with the Weibull distribution, the typical life of non-aging SAC305 under 16 MPa becomes 4639.SAC-Q resists fatigue more than SAC305 and SAC-R since its typical life is substantially longer.

Subsequently, individual SAC-Bi solder joints were aged for 10 and 1000 h at 125 ℃ and cycled until total failure under single-stress cycling conditions. After fitting the fatigue life of the seven individual solder joints with the Weibull distribution, the characteristic life of aged SAC305, SAC-Q, and SAC-R are presented in Table 5. The typical life of the same solder alloy and the aging period decreased as the stress amplitude increased. The typical life of the same solder alloy decreases as the aging period increases.

**Figure 6:**
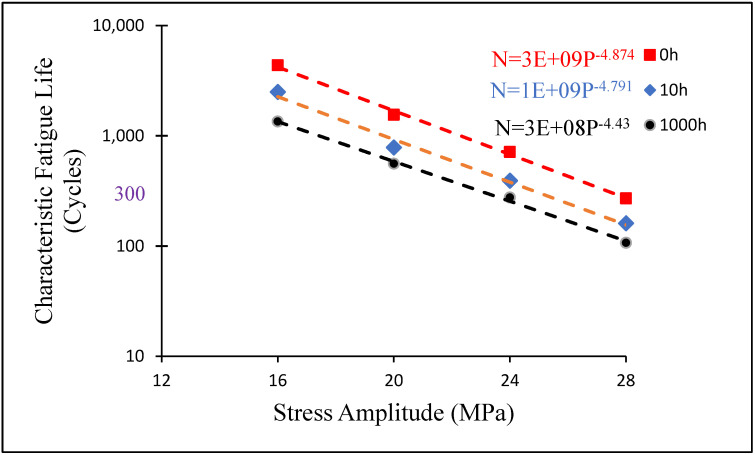
Fitted power equation of characteristic fatigue life (cycles) versus stress amplitude for non-aged, 10 h and 1000 h aged SAC305.

Based on the characteristic life (cycles) under a single stress amplitude, the fitted equations of a typical life for non-aged and aged SAC 305 on a log–log scale under four stress amplitudes are displayed in Figure 6. The fitted power equation, as shown in Equation (3), could be used to calculate the stress amplitude for any specified characteristic lifetime.

(3) $$N=a\times P^{-c}$$

where *N* is the typical fatigue life, *P* is the stress level, and a and c are the material constants. The mild and harsh stress amplitudes associated with 2500 and 300 cycles, respectively, were computed. A summary of mild and harsh stress amplitudes is presented in Table 2. For example, the typical life of non-aged SAC 305 under 18 MPa can attain 2500 cycles, whereas the typical life of SAC 305 under 27.6 MPa can reach 300 cycles. The same steps were implemented for the SAQ-Q and SAC-R to determine MS and HS.

**Figure 7:**
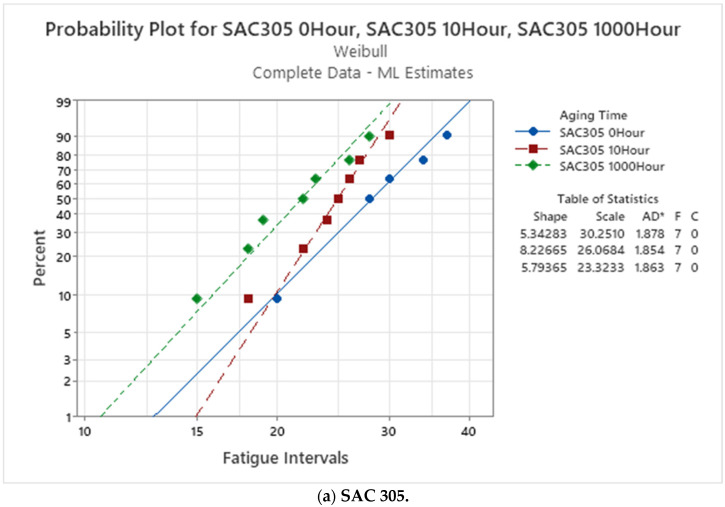

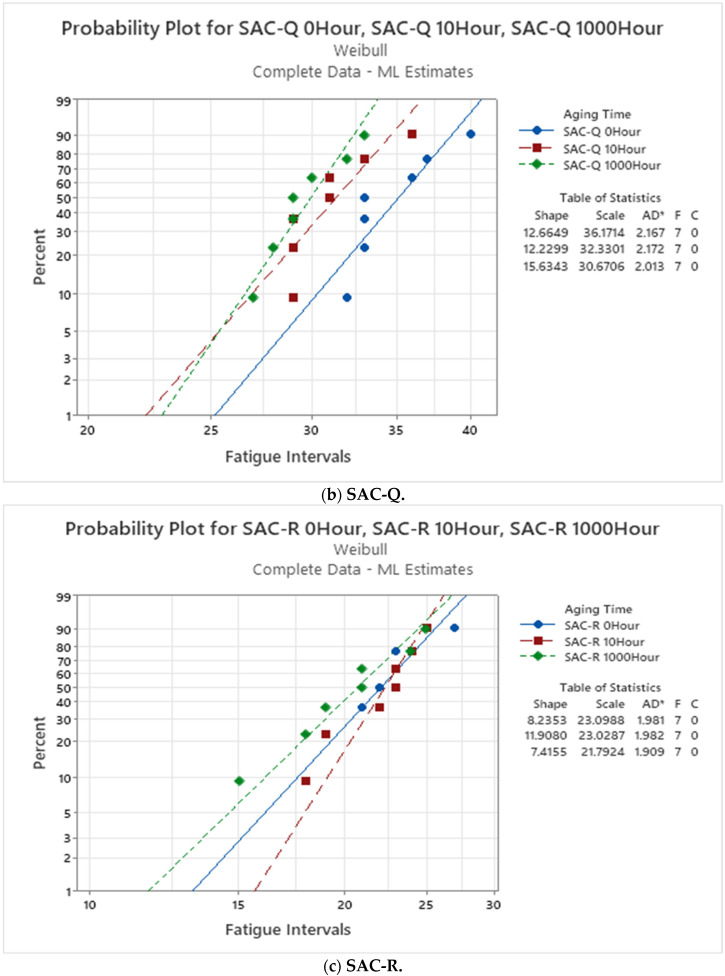
Fitted Weibull distribution and the characteristic fatigue life (intervals) of aged of (**a**) SAC 305; (**b**) SAC-Q; and (**c**) SAC-R in varying stress cycling.

Figure 7 shows the fitted Weibull maps for the non-aged, 10, and 1000 h of aging times for SAC305, SAC-Q, and SAC-R under varying stress amplitude tests. All the SAC305 samples failed before 50 intervals, as shown in Figure 7a. Consequently, more damage accumulated during varied stress amplitude testing than expected, and the SAC305 solder joint failed early. According to the test of the equal-scale parameter, the Weibull plot of SAC305 was considerably differentiated by the aging time, and the characteristic life (intervals) was significantly different. It can be observed that the typical lifetime decreases as the aging time increases. Furthermore, SAC305 declined with age in various stress amplitude tests because SAC305 showed less aging resistance at a single stress amplitude [28]. Similar degradation of SAC-Q was detected, as indicated in Figure 7b. In the equal-scale parameter test, the p-value was less than 0.05. As a result, with 95% confidence, the characteristic life of SAC-Q at non-aged, 10 h, and 1000 h aging times were considerably different. In addition, when the aging time increased, the SAC-Q typically decreased under varying stress amplitude testing. However, the scale parameter was not significantly different in the case of the SAC-R. Figure 7c depicts the fitted Weibull plots for SAC-R at non-aged, 10 h, and 1000 h of aging time under varied stress amplitude tests. It can be seen that the fitted Weibull plots of aged SAC-R were not substantially separated in the graph, and the characteristic life was close to each other. There is a diminishing trend in the characteristic life. However, this difference was not statistically significant.

By setting the characteristic life of the non-aged solder joint as the baseline, the reduced percentage of solder characteristic life can be computed after aging for 10 and 1000 h. SAC305 had the lowest aging resistance under varied stress amplitude testing. Compared with SAC305, SAC-Q displayed improved aging resistance in various stress cycles. In single stress cycles, SAC-Q exhibited the best aging resistance. However, the characteristic life of SAC-R after 10 h and 1000 h of aging did not reveal substantial degradation, decreasing by only 2% and 7%, respectively [28].

### 4.2. Work Dissipation Analysis

**Figure 8:**
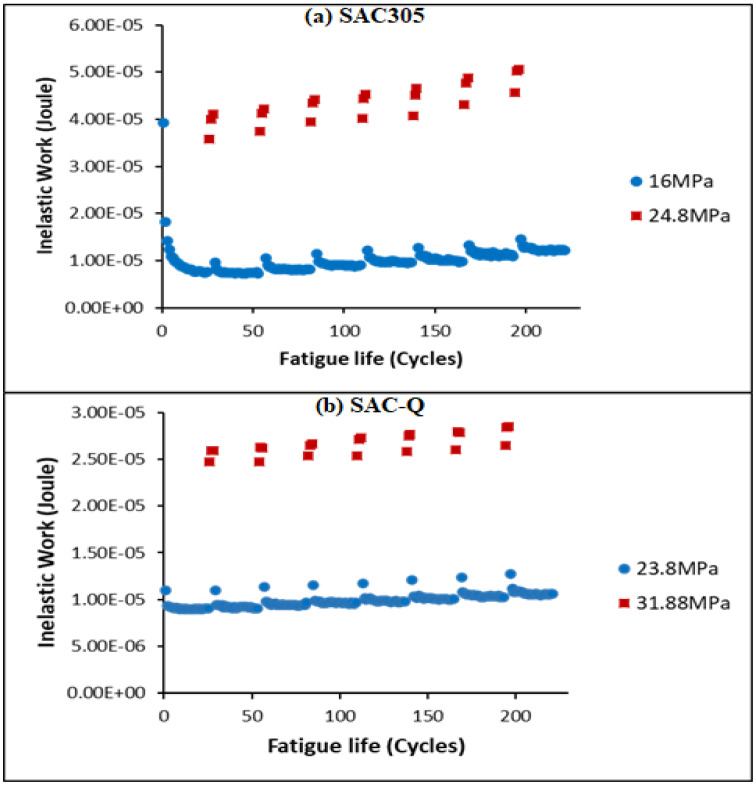
Example of inelastic work of (**a**) 10 h aged SAC305 under varying stress amplitude (16–24.8 MPa); (**b**) 1000 h aged SAC-Q varying stress amplitude (23.8–31.88 MPa).

Compared to non-aged SAC305, aged SAC305 performed similarly inelastic work after 10 and 1000 h. Figure 8a indicates the inelastic work performance of a 10 h aged SAC305 under 16–24.8 MPa. Owing to the flattening and hardening of the solder joint, the inelastic work dropped and hen but remained steady in the first 25 MS. Nonetheless, the inelastic work of both the MS and HS increased with each switch. This never reverts to the original level. Similar results were observed for aged SAC-Q and SAC-R. Figure 8b depicts a 10 h of an aged SAC-Q for inelastic work performance under varied 23.8–31.88 MPa cycling conditions. Before the first changeover, the inelastic work initially decreased, and then remained constant. The inelastic work increases dramatically when the stress amplitude is increased from 23.8 MPa to 31.88 MPa, which is reasonable considering that HS implies more damage every cycle. More significantly, there is an amplification in the level established prior to the 31.88 MPa cycles upon switching back to 23.8 MPa. This amplification of the inelastic work was observed throughout the life of individual solder joints with various stress amplitude cycles.

**Figure 9:**
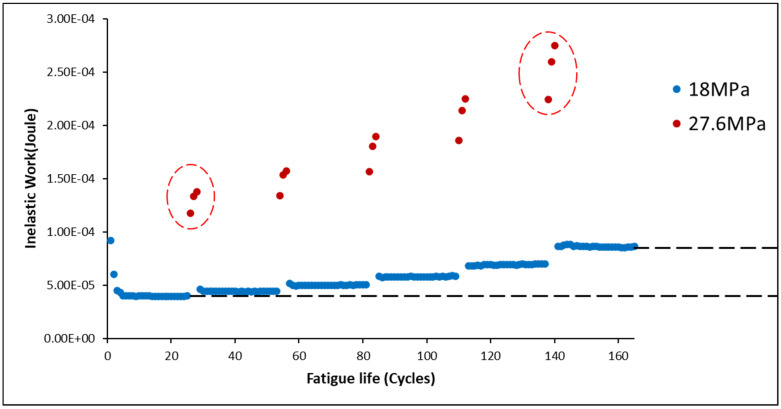
Inelastic work of non-aged SAC305 individual soler joint in varying stress amplitude test (18–27.6 MPa).

Figure 9 depicts the typical inelastic work performance of SAC305 at non-aged individual solder joints in the various stress amplitude tests. The inelastic work of the HS (27.6 MPa) cycles is represented by the red points, whereas the blue points indicate the inelastic work of the MS (18 MPa) cycles. The inelastic work of the MS cycles frequently leveled up after switching from HS to MS cycles. Cycle 160, for example, had significantly more inelastic work than cycle 20, even though the stresses in both cycles were the same. Meanwhile, as shown in Figure 9, the average inelastic work of the HS cycles increased after each changeover, as indicated by the red circles. Furthermore, the inelastic work of both the MS and HS was amplified after switching.

**Figure 10:**
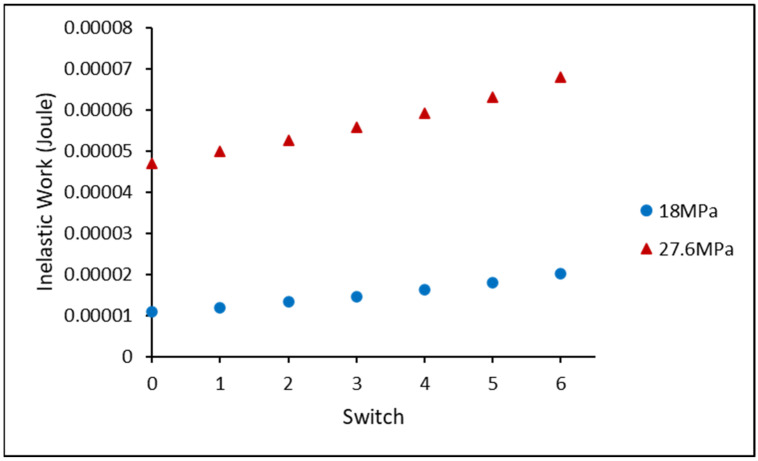
Average inelastic work of non-aged SAC305 individual soler joint in varying stress amplitude test (18–27.6 MPa).

**Figure 11:**
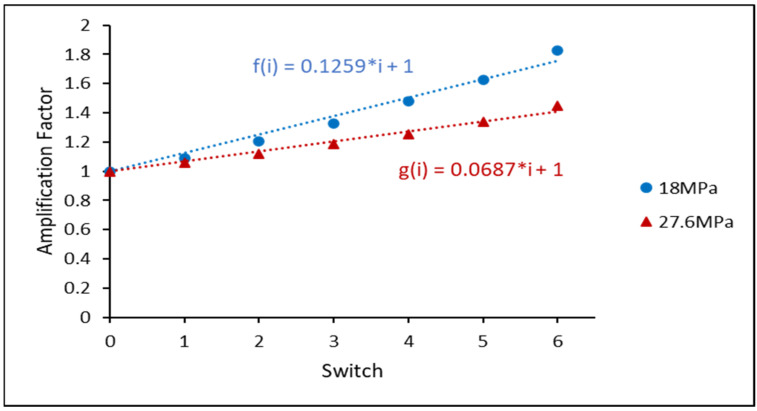
Amplification factor of non-aged SAC305 individual soler joint in varying stress amplitude test (18–27.6 MPa).

Figure 10 exhibits the average inelastic work for the non-aged SAC305 individual solder joints under varied stress amplitudes. The average inelastic work of the HS cycles (red triangles) and MS cycles (blue circles) for each switch was gathered to quantify the amplification phenomenon. As illustrated in Figure 11, the amplification factor is defined as the ratio of the inelastic work for each switch to the inelastic work before the first switch. The MS and HS amplification factors were effectively fitted using a linear regression. Furthermore, for the same solder joint, the slope of the linear equation of the HS cycle was lower than that of the MS cycle linear equation. It was revealed that, for non-aged SAC305, the inelastic work of the MS cycles increased faster than that of the HS cycles, independent of the solder alloy.

**Figure 12:**
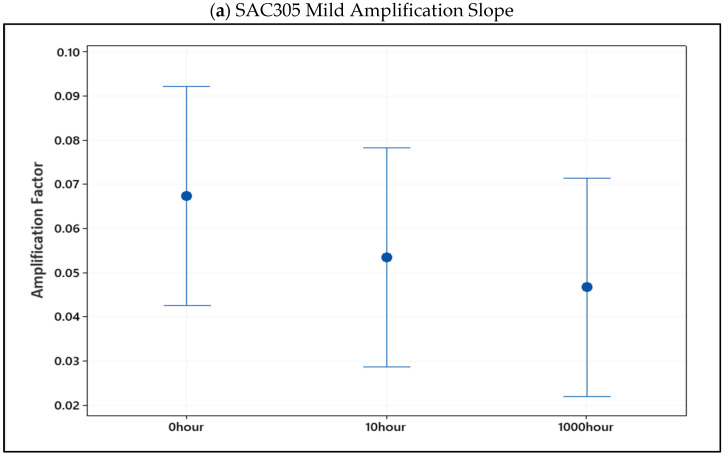

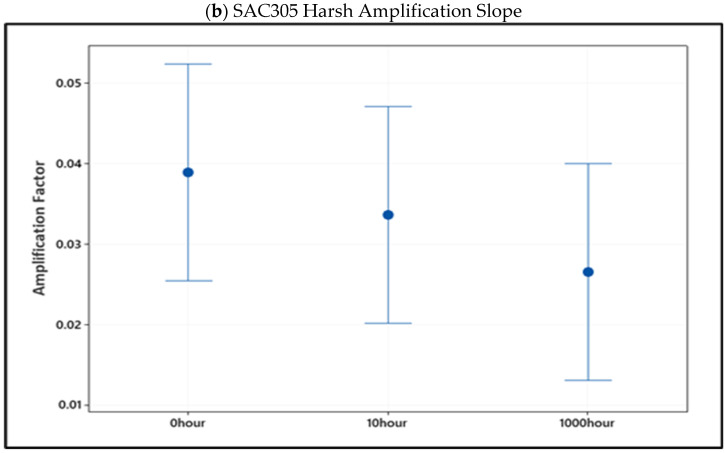
Confidence interval of (**a**) mild and (**b**) harsh amplification slope of non-aged, 10, and 1000 h aged SAC305 individual solder joint.

Figure 12a displays the 95% confidence range for the mild amplification slopes of SAC305 individual solder joints at non-aged, 10 h, and 1000 h. Even though there was a slight difference between the non-aged, 10, and 1000 h aging conditions, the average slope decreased as the aging period increased. As illustrated in Figure 12b, the harsh amplification slope is an excellent demonstration that SAC305’s inelastic work is less amplified with age.

**Figure 13:**
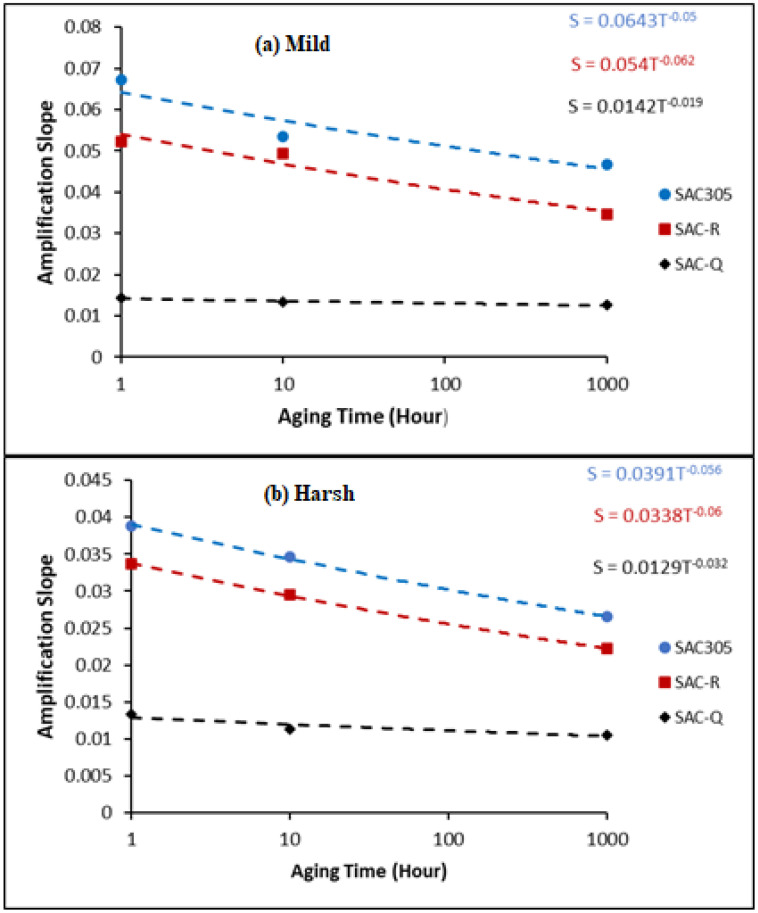
Power fit equation of average (**a**) mild and (**b**) harsh amplification slope versus aging time for SAC305, SAC-R, and SAC-Q.

Figure 13 illustrates the power fit equations of the average mild and harsh amplification slopes vs. aging times for SAC305, SAC-R, and SAC-Q. In general, SAC305 and SAC-R had a substantially larger mild amplification slope than that of SAC-Q. Consequently, in the cases of SAC305 and SAC-R, the inelastic work was amplified faster after each switch.

**Figure 14:**
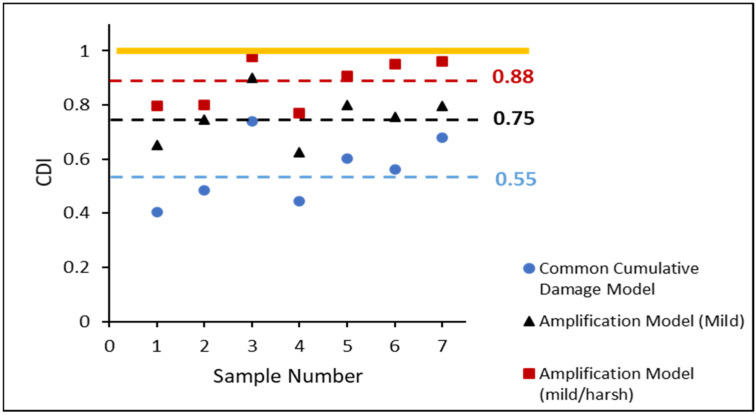
CDI of each totally failed non-aged SAC305 individual solder joint in varying stress amplitude test.

The estimated CDI of each entirely failed non-aged SAC305 solder joint in the various stress amplitude tests is displayed in Figure 14. The blue circles represent the estimates obtained using the CDA model. The calculated CDIs were significantly lower than 1, with an average of approximately 0.55, indicating that the samples failed completely. The black triangle is an estimate that considers the inelastic work of the MS. The accumulated damage estimation is improved to 0.75. When both mild and harsh amplification effects were considered, the average estimated CDI value was 0.88. However, there was a small gap in attaining a CDI value of 1, indicating that further work should be performed to optimize the CDI estimation.

**Figure 15:**
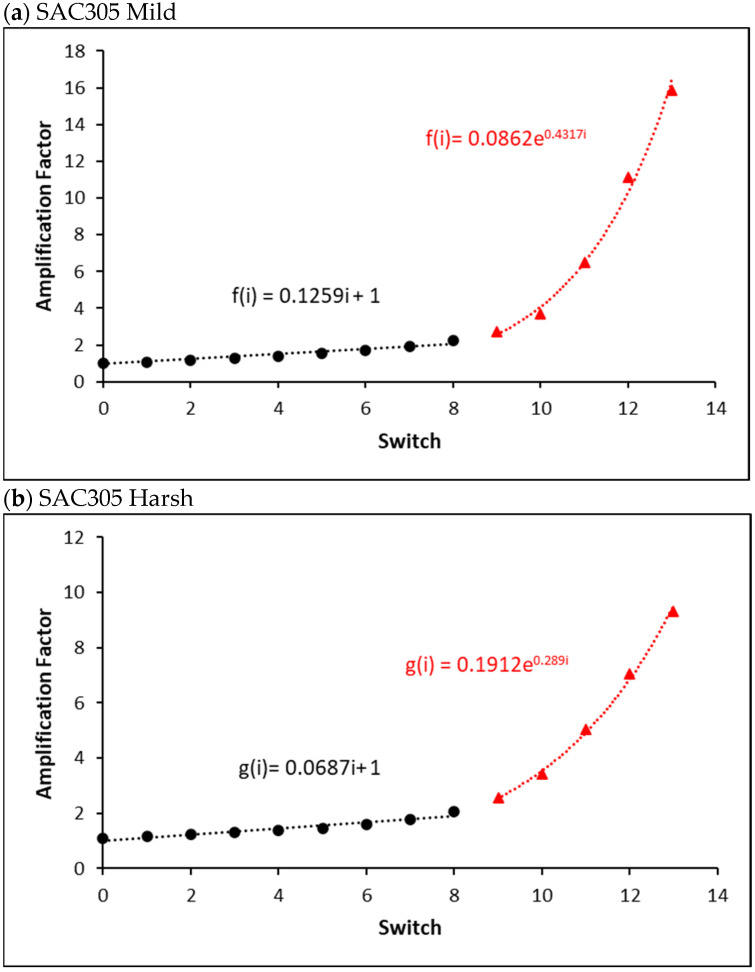

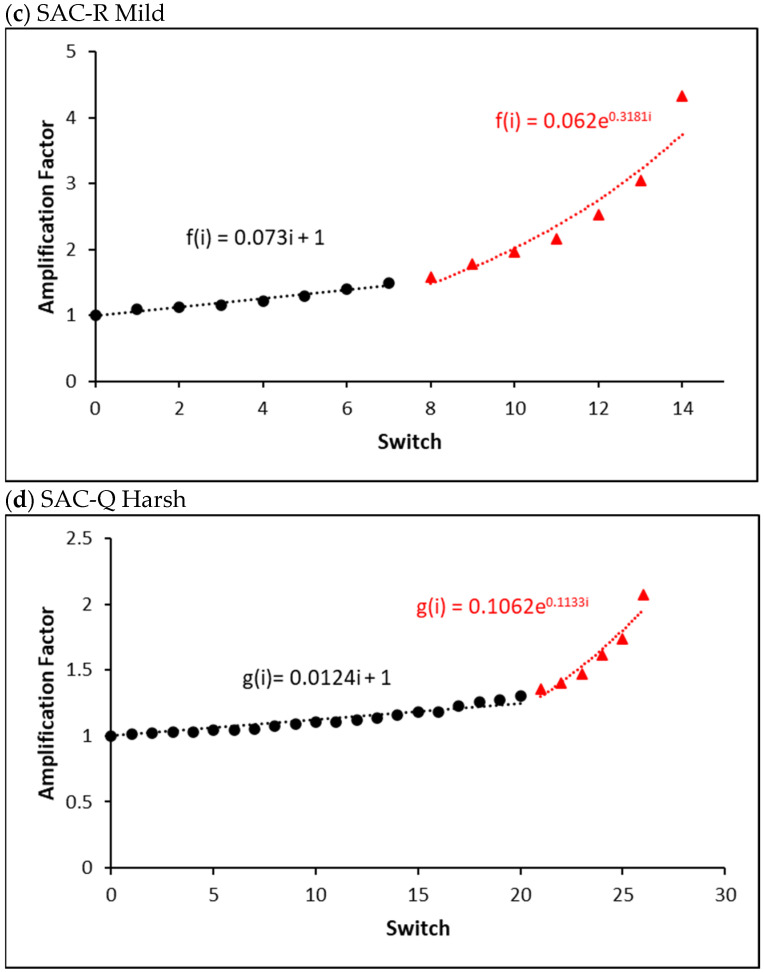
Mild and harsh amplification factor of non-aged (**a**) SAC 305 mild, (**b**) SAC 305 harsh, (**c**) SAC-R mild, and (**d**) SAC-Q harsh individual solder joint in varying stress amplitude test.

Figure 15a,b depict the mild and harsh amplification factors without aging for the SAC305 individual solder connection under various stress amplitude conditions. In the early stages of the fatigue test, inelastic work was linearly enhanced after each switch. However, after a certain number of switches, the inelastic work increased until the utter failure. Consequently, under various stress amplitude conditions, a piecewise function was employed to explain the inelastic work performance of the individual solder joints until total failure. The linear regression equations (black circles) estimate the damage accumulated before the 9th switch, as shown in Figure 15a,b. Exponential equations (red triangles) are used until failure.

Figure 15c depicts a typical example of the mild amplification factor of a non-aged SAC-R individual solder joint under various stress amplitude settings. Similar piecewise functions are also fitted. Damage accumulation was assessed using a linear function for the entire fatigue life of the tested solder joints under stress cycling using the previously suggested CDI calculation methodology. However, in the last several switch counts, the damage was amplified faster than predicted, and the linear function from switches 8 to 14 underestimated it.

**Figure 16:**
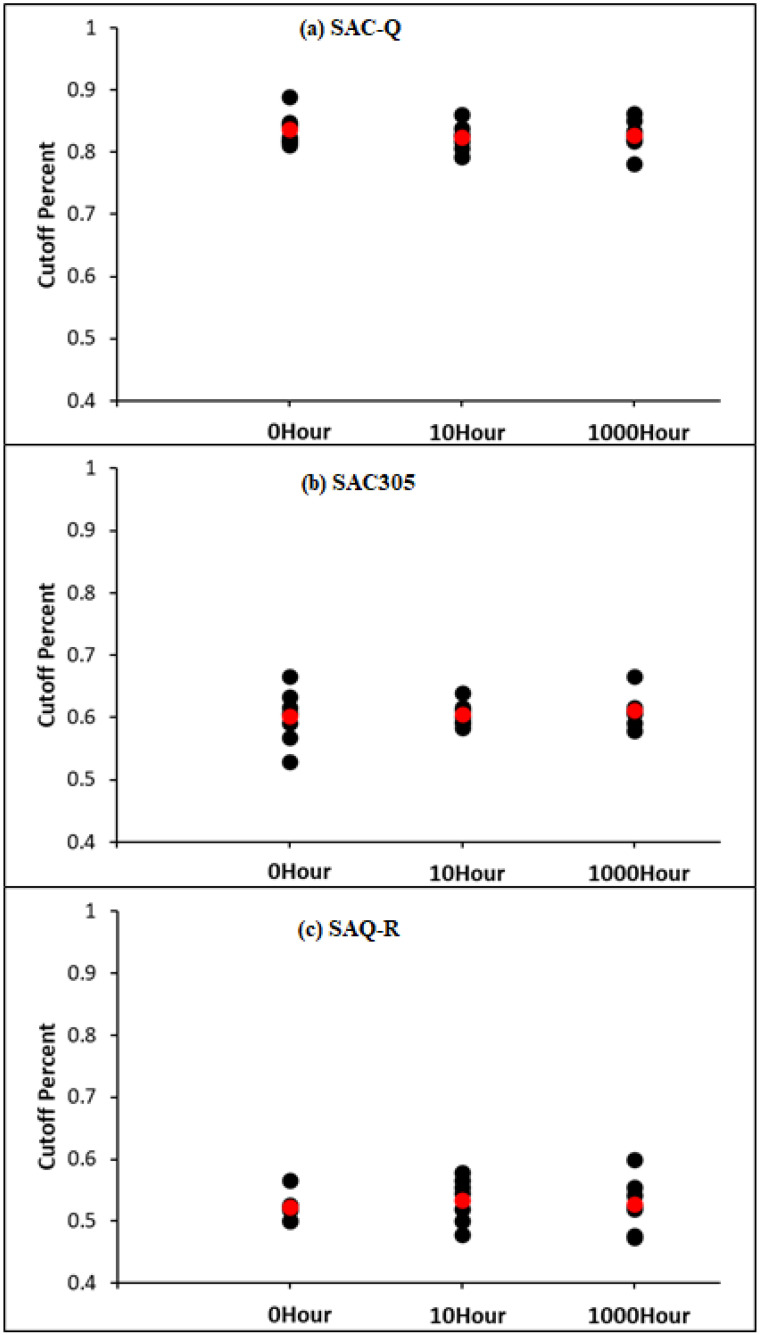
Cut-Off summary for non-aged, 10, and 1000 h aged for (**a**) SAC-Q, (**b**) SAC305, and (**c**) SAC-R individual solder joint.

A typical example of the harsh amplification factor of a non-aged SAC-Q individual solder joint under various stress amplitude conditions is shown in Figure 15d. A similar piecewise function is used to fit the amplification factor. However, compared with SAC305 and SAC-R, the percentage of linear components was substantially higher. The key is determining the cut-off point for various solder joints. The cut-off summary for the non-aged, 10, and 1000 h aged SAC-Q solder joints is shown in Figure 16a. The cut-off value represents the percentage of linear amplification. The graph shows that the typical cut-off point (red) is approximately 83%, indicating that under various stress amplitude conditions, inelastic work linearly magnifies approximately 83% of an individual solder joint’s overall life. The amplification factor increases exponentially during the last 17% of the overall lifespan. There is no strong indication that the aging time influences the cut-off points in the case of SAC-Q individual solder joints at a 5% significance level. The cut-off summary for the non-aged, 10, and 1000 h aged SAC305 and SAC-R individual solder joints is shown in Figure 16b,c. In general, the average linear cut-off percentages of SAC305 and SAC-R were lower than that of SAC-Q. Similarly, there is no evidence to support a 5% significance level of proof that aging time influences the cut-off points.

### 4.3. Proposal Model

A refinement of the modified Miner model was performed using Equations (4)–(6) [22], in which the amplification factor is divided into initial and steady amplifications.

(4) $$1=\overset{s}{\underset{i}{\sum }}\left\{f{\left(i\right)}_{in}\frac{n_{in}}{N_{m}}+f{\left(i\right)}_{st}\frac{n_{mi}-n_{in}}{N_{m}}+\frac{n_{hi}}{N_{h}}\right\}$$

with

(5) $$f{\left(i\right)}_{in}=\alpha i+b_{in}$$

and

(6) $$f{\left(i\right)}_{st}=\alpha i+1$$

where $f{\left(i\right)}_{in}$ refers to the amplification function in the initial state, $f{\left(i\right)}_{st}$ is the amplification function in the steady-state, α represents the slope of the amplification function, $b_{in}$ refers to the amplification constant in the initial state, $n_{in}$ indicates the number of cycles in the initial state, $n_{mi}$ refers to the number of cycles at low amplitude, $N_{m}$ represents the life at low amplitude, $n_{hi}$ is the number of cycles at high amplitude, $N_{h}$ indicates the life at high amplitude, and i is the number of intervals. A higher amplitude value was reported to result in a steeper amplification slope for the SAC305 alloy [22]. In addition, with high amplitude cycle counts in each set, the slope of amplification in SAC305 increased, but it eventually leveled off [29]. Furthermore, the work amplification value at lower amplitudes is influenced by the sequence of larger amplitudes preceding the lower stress amplitude [7].

Based on the above analyses, we propose our final damage accumulative model as follows:

(7) $$CDI=\underset{i}{\sum }f\left(i\right)\frac{n_{m}}{N_{m}}+g\left(i\right)\frac{n_{h}}{N_{h}}$$

where CDI is the common damage index, $n_{m}$ refers to the number of cycles under MS amplitude, $N_{m}$ is the number of cycles when a failure occurs under MS amplitude, $n_{h}$ represents the number of cycles under HS amplitude, $N_{h}$ refers to the number of cycles when a failure occurs under HS amplitude, and f(i) and g(i) are the amplification functions, that represent the effect of the switch on the inelastic work amplification for MS and HS cycles. In addition, the amplification function is defined by a piecewise function consisting of linear and exponential functions.

**Figure 17:**
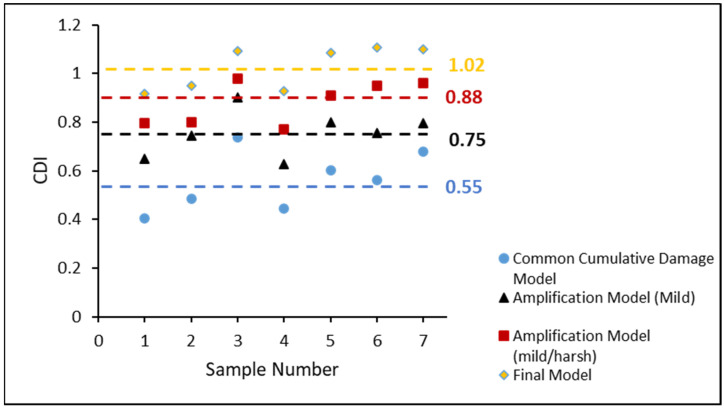
CDI comparison of non-aged SAC305 individual solder joint in varying stress amplitude test.

Figure 17 compares the accumulative damage models in the case of non-aged SAC305 individual solder joints in the varying stress amplitude tests. Using the CDA model, the blue circles indicate the expected CDI of each non-aged SAC305 solder joint. The black triangles (linear function) show the CDI predicted using the MS cycles amplification function indicated by the black triangles (linear function). The red squares indicate the CDI predicted using the amplification function (linear function) of the MS and HS cycles. The CDI predicted is shown by yellow diamonds, which consider the amplification function (piecewise function) of the MS and HS cycles. Compared to previous models, the suggested model’s average CDI was extremely close to 1.

### 4.4. Microstructure Analysis

**Figure 18:**
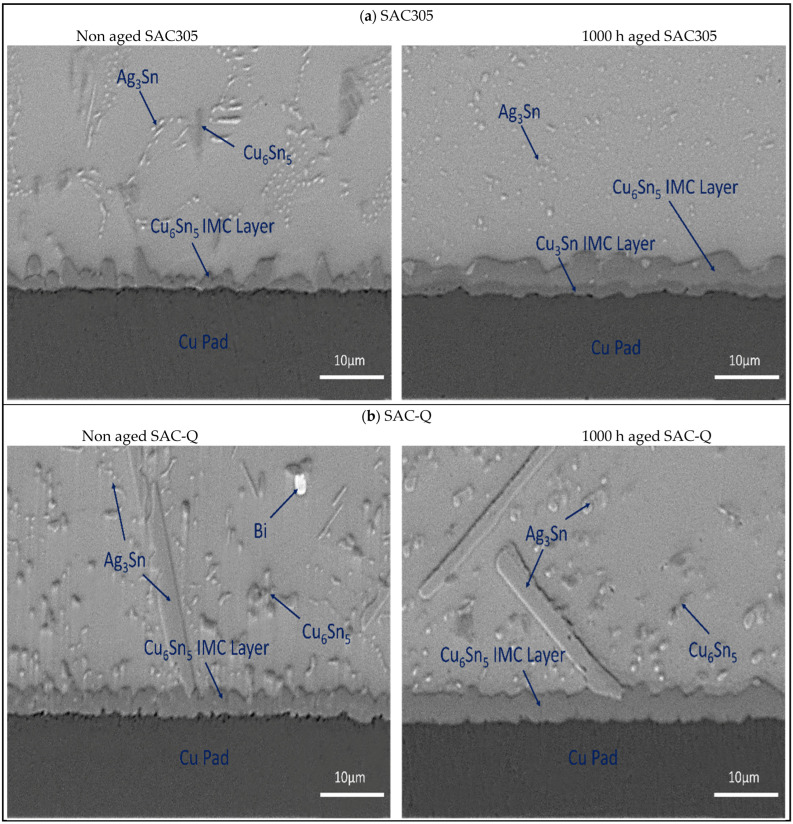

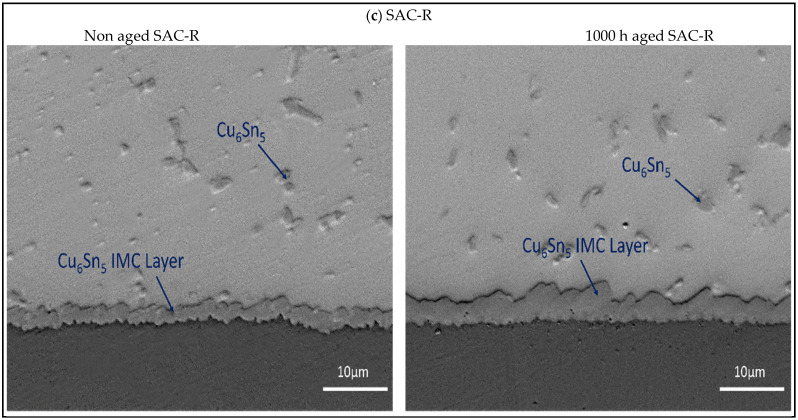
Comparison of aging effect on interface area for (**a**) SAC305, (**b**) SAC-Q, and (**c**) SAC-R.

Figure 18a–c depict the SEM microstructure of the Cu pad/solder alloy interface region, including a comparison of the non-aged and 1000 h aged interfaces. As displayed in Figure 18a, the thickness of the IMC layer almost doubled from 1.8 μm to 3.7 μm. A new IMC layer (Cu_3_Sn) was also formed between the Cu pad and Cu_6_Sn_5_. This growth of the Cu_3_Sn sub-layer increased the total IMC thickness, increasing the chance of brittle fractures owing to micro-voids formation inside the interface, which is detrimental to fatigue characteristics. In contrast, little IMC layer development was detected in the SAC-Q solder joint. The thickness was measured as approximately 3 μm. The IMC thickness of the SAC-R solder alloy increased significantly from 1.8 μm to 2.8 μm. By preventing Sn from diffusing into the IMC layer, the inclusion of Bi suppressed the growth of the IMC and the density of cavities during aging.

**Figure 19:**
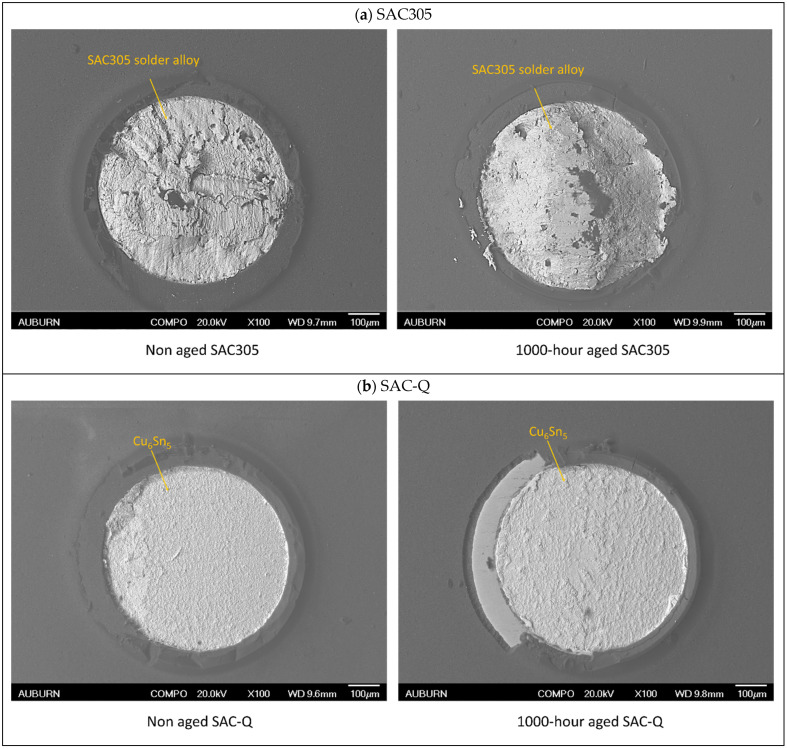

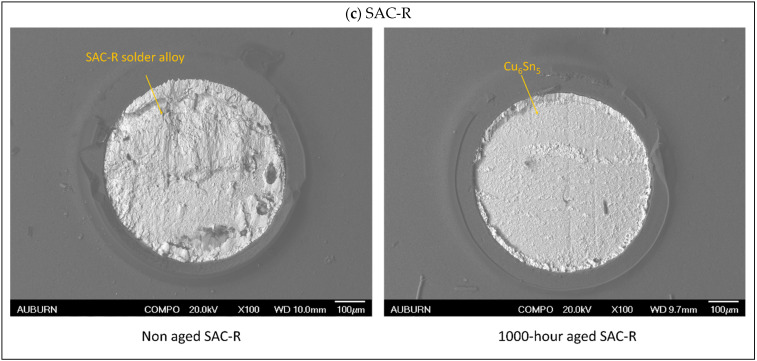
Comparison of aging effect on failure modes for (**a**) SAC305, (**b**) SAC-Q, and (**c**) SAC-R.

The top views of the fractures for SAC-305, SAC-Q, and SAC-R are demonstrated in Figure 19a–c, respectively. After the fatigue test for SAC305, portions of the solder alloy, predominantly Sn, remained and covered the Cu pad. The presence of Sn was detected using EDS analysis, indicating a ductile failure mode. This observation was in line with other studies, where the SAC305 with OSP surface failure mechanism was studied [6,30]. In this case, the cracks propagated throughout the bulk solder. The same ductile failure mode was observed for SAC305 with and without aging. In the top-view image of SAC-Q, a distinct separation between the bulk solder and IMC layer indicates brittle failure. The IMC layer became coarser with aging. Once the SAC-R solder alloy was aged for 1000 h, its failure mechanism changed from ductile to brittle. The absence of Ag during aging accelerated the coarsening rate of the Cu_6_Sn_5_ grains. Consequently, the fractured surface morphology was ductile, with massive Cu_6_Sn_5_ grains in the bulk solder joint.

**Figure 20:**
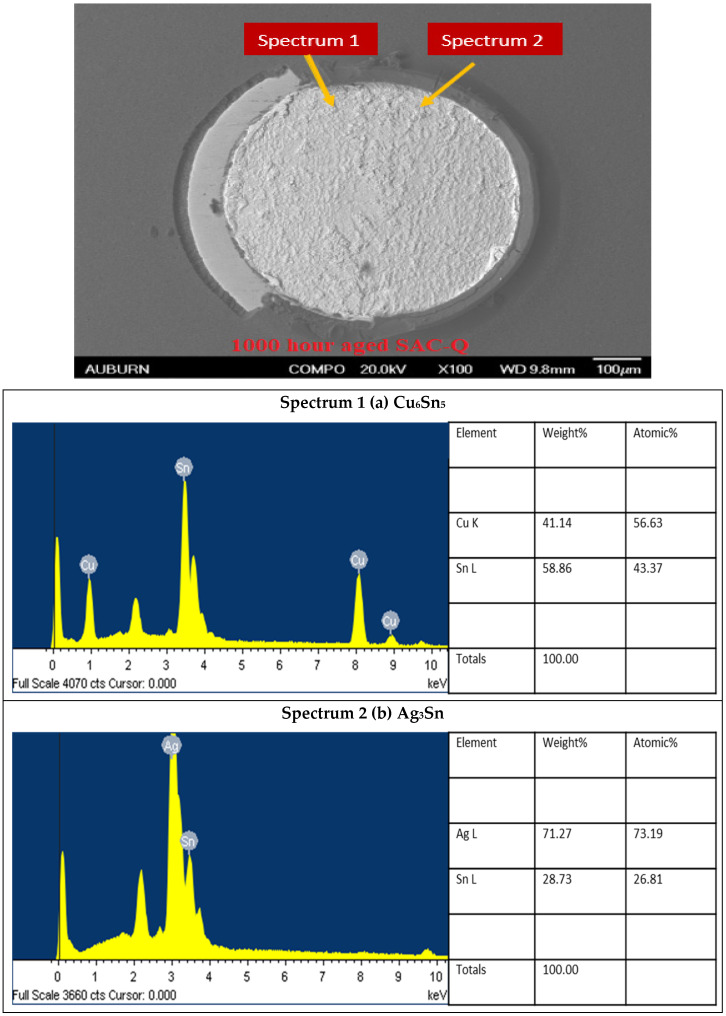
(**a**) Energy dispersive X-ray (EDS) spectrum analysis of Cu_6_Sn_5_, (**b**) Energy dispersive X-ray (EDS) spectrum analysis of Ag_3_Sn.

It is worth noting that the EDS analysis was performed to identify each phase separately by acquiring and analyzing data for each spectrum separately. Furthermore, using electron backscatter diffraction (EBSD), we can distinguish between the IMC precipitate since Ag (47: atomic number) has brighter contrast compared to Cu (27: atomic number). Energy dispersive X-ray (EDS) spectrum analysis of Cu_6_Sn_5_ and Ag_3_Sn was performed for each alloy after reflow and at 1000 h of aging times, as indicated in Figure 20.

## 5. Conclusions

The fatigue performance of aged SAC305, SAC-Q, and SAC-R was studied under varying stress cycles. In variable stress amplitude testing, a novel damage accumulation model was suggested to assess the CDI of SAC-Bi individual solder joints. Amplification of inelastic work was observed for all solder joints and aging periods after each change between the MS and HS cycles. This study focused on the amplification functions of several solder alloys under various aging conditions. A novel damage accumulative model was presented in variable stress amplitude testing to assess the CDI of SAC-Bi individual solder junctions. It was compared to another cumulative damage model and offered a better estimate of CDI than other models. The results are summarized as follows:

SAC305, SAC-Q, and SAC-R failed earlier than the standard cumulative damage model predicted in the variable stress amplitude tests.

The typical life of SAC305, SAC-Q, and SAC-R diminished after 10 h and 1000 h of aging.

SAC-Q outperformed SAC305 and SAC-R in terms of fatigue resistance in variable-stress amplitude testing.

Ductile failure mechanisms are often observed in varying stress tests of SAC305, regardless of the aging conditions. In contrast, the SAC-Q solder alloy with the OSP surface finish exhibited brittle surface morphology under variable stress amplitude tests. The absence of Ag in SAC-R was prominent in causing ductile fracture during aging owing to the coarsening of the IMC precipitates.

In the varied stress amplitude tests, the aging time did not affect the failure mode of the individual solder joints.

A “raise-up” phenomenon of inelastic work was identified in variable stress amplitude experiments after transitioning between moderate and hard stress cycles. Furthermore, switching amplifies the inelastic work at the equivalent stress cycles. Consequently, an amplification factor was suggested in the cumulative damage model.

The HS cycles had a steeper amplification slope than the MS cycles. The amplification slope tended to decrease when the solder joint was aged for a longer period.

The amplification factor increased linearly in the early cycling stages, but rapidly increased beyond the predetermined switch counts. As a result, a piecewise function was suggested to measure the inelastic work amplification.

The SAC305 and SAC-R amplification factors were linearly increased until they reached 50–60% of their fatigue life. Subsequently, it began to expand until a failure occurred. On the other hand, the SAC-Q has an 80–90% cut-off percentage of its fatigue life.

“Under singles stress amplitudes, at 24 and 28 MPa, SAC-Q solder joints are proven to be more durable, followed by SAC-R, than SAC305, regardless of the aging conditions. After reflow, the fatigue life of AC-R and SAC305 represented 28% and 22% of the overall life for SAC-Q, respectively. After 1000 h of aging, SAC-R and SAC305 exhibited 77% and 87% of SAC-Q’s characteristic life, respectively.

Under varying stress amplitudes, SAC305 had the lowest aging resistance under varied stress amplitude testing. SAC-R has been proven to degrade less after 10 and 1000 h of aging by showing 2% and 7%, respectively. However, SAC-Q showed the best fatigue life performance. The proposed model fits the best SAC305 system as compared to other alloy systems.”

## Figures and Tables

**Table 1:** Composition of the solder materials.

Alloy	Composition
SAC305	Sn (96.5%), Ag (3%), Cu (0.5%)
SAC-Q	Sn 92.77%, Ag 3.41%, Cu 0.52%, Bi 3.3%
SAC-R	Sn (96.62%), Cu (0.92%), Bi (2.46%)

**Table 2:** Stress amplitude for solder alloys aged for 0, 10, 1000 h.

Alloy	Aging Time (h)	Stress Amplitude	
		MS (MPa)	HS (MPa)
SAC305	0	18	27.6
	10	16	24.8
	1000	14.4	23.48
SAC-Q	0	24	32
	10	23.8	31.88
	1000	23.6	31.72
SAC-R	0	17.5	28.9
	10	17.08	28.48
	1000	16.8	28

**Table 3:** Varying amplitude test plan of aged solder alloys aged for 0, 10, 1000 h.

Alloy	Aging Time (h)	Test Matrix	
		Test Method	Sample Test
SAC305	0	25 MS cycles + 3 HS cycles	7
	10	25 MS cycles + 3 HS cycles	7
	1000	25 MS cycles + 3 HS cycles	7
SAC-Q	0	25 MS cycles + 3HS cycles	7
	10	25 MS cycles + 3 HS cycles	7
	1000	25 MS cycles + 3 HS cycles	7
SAC-R	0	25 MS cycles + 3 HS cycles	7
	10	25 MS cycles + 3 HS cycles	7
	1000	25 MS cycles + 3 HS cycles	7

**Table 4:** Characteristic life (cycles) of non-aged individual solder joints.

	Stress Amplitudes (MPa)					
	16	20	24	28	32	36
SAC305 (cycles)	4369	1551	713	271	-	-
SAC-Q (cycles)	-	-	2493	888	303	133
SAC-R (cycles)	2965	1308	560	375	-	-

**Table 5:** Characteristic life (cycles) of aged individual solder joints.

	Aging Time (h)	Stress Amplitudes (MPa)					
		16	20	24	28	32	36
SAC305 (cycles)	10	2501	785	392	161	-	-
	1000	1350	559	276	107	-	-
SAC-Q (cycles)	10	-	-	2259	851	290	129
	1000	-	-	2099	835	278	122
SAC-R (cycles)	10	2762	1189	538	356	-	-
	1000	2656	1092	511	304	-	-

## Data Availability

The authors confirm that the data supporting the findings of this study are available within the article or upon request from the corresponding author.

## Nomenclatures

**List of Abbreviations**		**List of Symbols**	
Ag	Silver	a and c	Material constants
Bi	Bismuth	$b_{in}$	Amplification constant in the initial state
CDA	Common damage accumulation	f(i), g(i)	Amplification function
CDI	Cumulative damage index	$f{\left(i\right)}_{in}$	Amplification function in the initial state
HS	Harsh stress	$f{\left(i\right)}_{st}$	Amplification function in the steady-state
ImAg	Immersion silver	$n_{hi}$	Number of cycles at high amplitude
IMC	Intermetallic compound	$n_{i}$	Number of cycles
In	Indium	$n_{in}$	Number of cycles in the initial state
MS	Mild stress	$n_{mi}$	Number of cycles at low amplitude
Ni	Nickel	$N_{h}$	Life at high amplitude
OSP	Organic solderability preservatives	$N_{m}$	Life at low amplitude
PBGA	Plastic ball grid array	P	Stress level
PCB	Printed circuit board	α	Slope of the amplification function
SAC	Tin-silver-copper (SnAgCu)		
Sb	Antimony		
SEM	Scanning electron microscope		
Sn-Pb	Tin-lead		
